# Synergistic Aggregation-Induced Emissive Linkers in Metal–Organic Frameworks for Ultrasensitive and Quantitative Visual Sensing

**DOI:** 10.1021/jacsau.5c00092

**Published:** 2025-03-17

**Authors:** Yansong Jiang, Wenxin Chang, Zhihao Li, Xiang Zhou, Panjing Zhang, Xuehai Huang, Xinyi Pan, Zhenda He, Yu Wang, Zhongqun Tian

**Affiliations:** † South China Advanced Institute for Soft Matter Science and Technology, School of Emergent Soft Matter, South China University of Technology, Guangzhou 510640, China; ‡ State Key Laboratory of Physical Chemistry of Solid Surfaces and College of Chemistry and Chemical Engineering, Xiamen University, Xiamen 361005, China; § Center for Electron Microscopy, South China University of Technology, Guangzhou 510640, China; ∥ Guangdong Provincial Key Laboratory of Functional and Intelligent Hybrid Materials and Devices, South China University of Technology, Guangzhou 510640, China

**Keywords:** luminescent MOF, AIE, visual sensor, pesticide detection, quantitative sensing

## Abstract

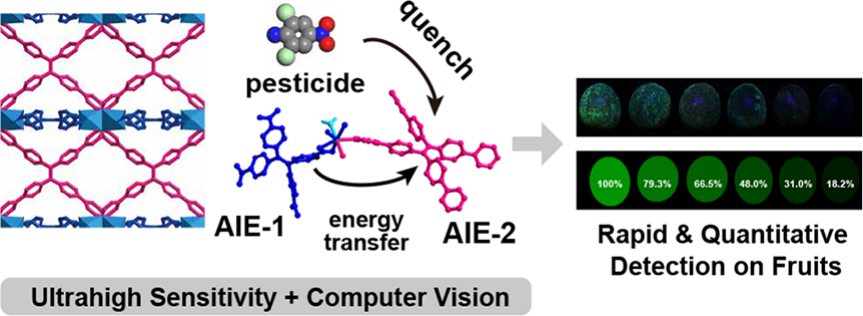

Luminescent metal–organic frameworks (MOFs) represent an emerging class of materials for visual analyte detection. In this study, we present a strategy that integrates two synergistic aggregation-induced emissive (AIE) linkers into a MOF, significantly enhancing sensing sensitivity, selectivity, and quantification capabilities for practical applications. The dual AIE linkers simultaneously optimize porosity and amplify emission intensity. The tailored pore structure precisely matches the molecular dimensions of the pesticide 2,6-dichloro-4-nitroaniline (DCN), while Förster resonance energy transfer between the linkers achieves an exceptional fluorescence quantum yield of 92.6%. This design enables ultrasensitive DCN detection in water, with an unprecedented detection limit at the ppb level, along with superior selectivity, rapid response time, high quantification accuracy, recyclability, and strong resistance to interference. A comprehensive investigation using UV–vis, fluorescence, transient absorption, X-ray photoelectron, and Raman spectroscopies, supported by theoretical calculations, attributes the efficient fluorescence quenching to photoinduced energy transfer. Additionally, we demonstrate instant, naked-eye detection of DCN residues on fruit surfaces and contaminated soil by applying MOF solutions and illuminating under UV light. Quantitative analysis of DCN residues on fruits was further achieved using computer vision and a custom script, providing a practical, on-site method for rapid and precise detection of pesticide residues.

## Introduction

Metal–organic frameworks (MOFs), a class of inorganic–organic hybrid solids,^1^ have gained wide application in areas such as adsorption and separation,^2−4^ catalysis,^5,6^ fluorescent sensing,^7−11^ and magnetism.^12^ Among these, luminescent MOFs (LMOFs) have emerged as promising materials for various analytical and bioanalytical applications.^13−15^ LMOFs offer two key advantages: their well-defined, tunable pore structures and their ability to incorporate functional units, making them ideal platforms for designing molecular recognition systems for specific analytes.^16−20^ To date, LMOF-based sensors have been applied in detecting heavy metals, pesticides, organic pollutants, and biomolecules.^21−28^

Compared to conventional analytical techniques such as mass spectrometry (MS) and high-performance liquid chromatography (HPLC), LMOF-based sensors provide sufficient sensitivity and selectivity while offering additional benefits like rapid response, cost-effectiveness, and ease of use.^7,8,29^ For example, Tang et al. developed a series of Ln-MOFs with limits of detection (LOD) for antibiotics below 1 μM.^30^ Liu et al. reported a calix[4]arene-decorated 2D MOF nanosheet for glyphosate detection with LOD of 2.25 μM.^31^ Fedin et al. demonstrated a highly selective Ln-MOF for gossypol detection, which was unaffected by blood plasma and urine samples,^32^ and Lang et al. introduced a quick-responsive Cu-MOF that detected volatile organic compounds with a response time of less than one second for CH_2_Cl_2_.^33^ These remarkable sensing properties position LMOFs as strong candidates for rapid on-site chemical evaluations.

However, integrating all these desirable attributes into a single LMOF remains a significant challenge, particularly when aiming at quantitative performance comparable to MS and HPLC.^8^ Thus, developing strategies that combine the advantages of LMOFs with enhanced quantification is crucial for advancing MOF sensors toward real-world applications.

In this work, we present a strategy that enhances LMOF sensors by incorporating two synergistic aggregation-induced emissive (AIE) linkers. This approach demonstrates excellent performance in pesticide detection, showcasing its potential for practical applications. Compared to LMOFs with non-AIE or single AIE ligands, dual-AIE LMOFs exhibit improved fluorescence intensity due to interlinker interactions, along with greater flexibility in tuning porosity to target specific analytes. As a result, the dual-AIE LMOFs deliver exceptional performance in visibility, sensitivity, stability, selectivity, and resistance to interference. We demonstrate the practical potential of this system for rapid on-site pesticide detection in fruits and soil, enabling naked-eye qualitative detection and unprecedented quantitative analysis via computer vision.

## Results and Discussion

### Design, Synthesis, and Structures of MOFs

The synergistic linkers used in this study are two tetrahedral aggregation-induced emission (AIE) ligands: 4,4′,4″,4‴-(ethene-1,1,2,2-tetrayl)tetrabenzoic acid (H_4_TCPE) and 1,1,2,2-tetrakis(4-(pyridin-4-yl)phenyl)-ethene (TPPE). Incorporating these linkers together into metal–organic frameworks (MOFs) offers several advantages: (1) introducing novel framework topologies through the complementary sizes and coordination groups of the two linkers, (2) enhancing AIE fluorescence via interlinker energy transfer, and (3) improving selectivity due to the diverse aromatic units and coordination groups present. Using these linkers with Cd or Zn ions, we synthesized four representative MOFs via one-pot solvothermal reactions (see SI for details): **1** (Cd_2_(TCPE)(H_2_O)_4_), **2** (Cd(TPPE)Cl_2_), **3** (Cd_2_(TCPE)(TPPE)(H_2_O)_2_), and **4** (Zn_2_(TCPE)(TPPE)).

**Figure 1:**
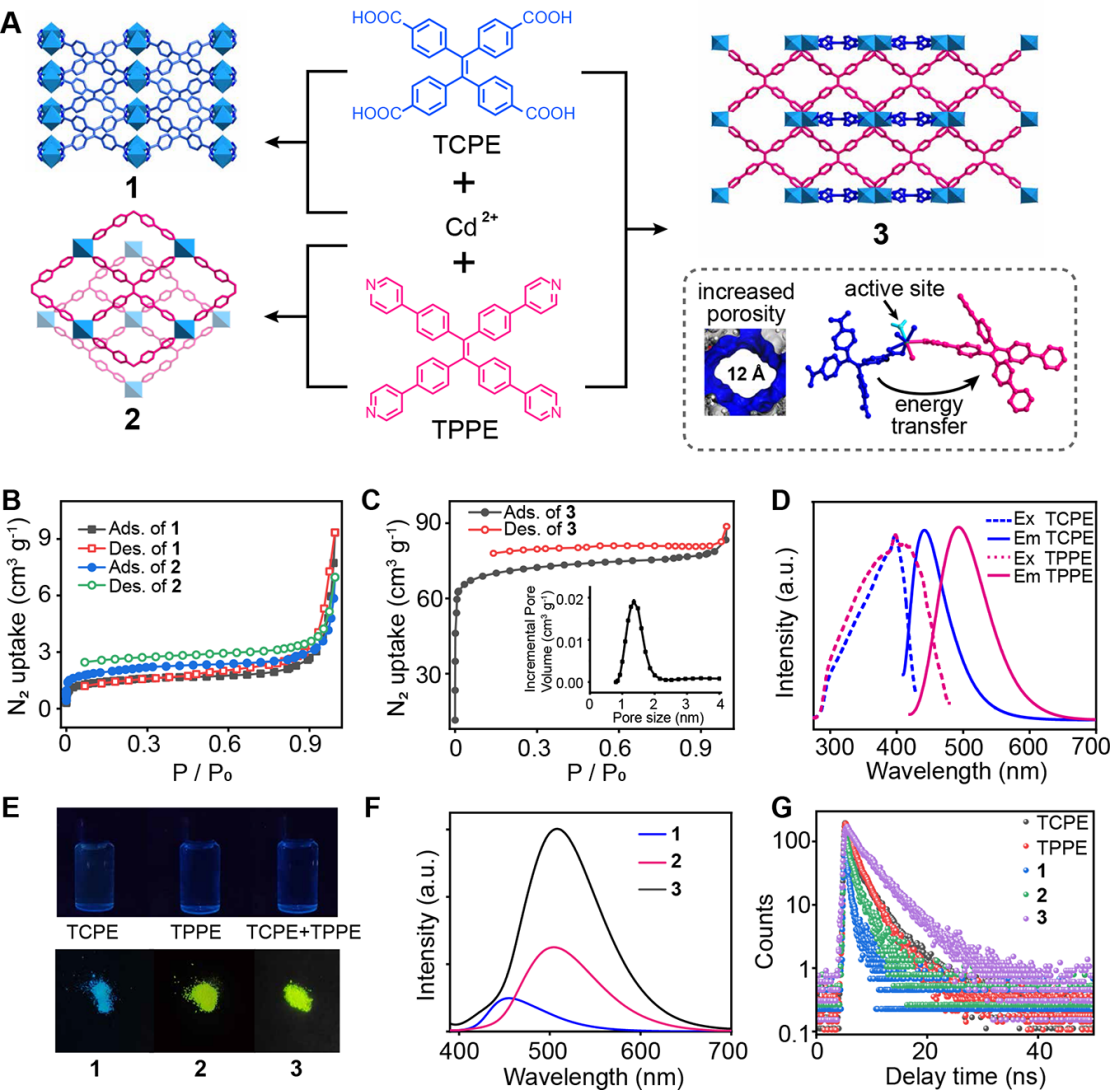
Metal–organic frameworks (MOFs) incorporating two synergistic aggregation-induced emissive (AIE) linkers. (A) Synthetic routes and single crystal structures of compounds **1**–**3**. The diagram illustrates the benefits of integrating two AIE ligands: simultaneous control of pore size and enhancement of fluorescence properties through interligand energy transfer. (B) N_2_ adsorption isotherms of **1** and **2** at 77 K. (C) N_2_ adsorption isotherm and the pore size distribution (inset) of **3** at 77 K. (D) Fluorescence excitation and emission spectra of TCPE and TPPE monomers. (E) Photographs of ligand solutions (1 mg·mL^–1^) and MOF solids under 365 nm UV light. (F) Fluorescence emission spectra of **1**–**3** solids. (G) Fluorescence lifetimes of ligands and MOFs.

Single-crystal structure analysis reveals that the dual-linker MOFs **3** and **4** exhibit greater porosity than the single-linker MOFs **1** and **2**. MOF **1** crystallizes in the orthorhombic *Pbam* space group. Each Cd center coordinates with six oxygen atoms, four from two carboxylate groups and two from coordinated water molecules (Figure S1). These Cd ions and carboxylate groups form a one-dimensional −Cd(COO)– chain, and each TCPE ligand connects four chains, creating a three-dimensional interpenetrated framework (Figure 1A).

MOF **2** crystallizes in the orthorhombic system *I*222 space group. Each Cd center is coordinated with four pyridine nitrogen atoms from TPPE and two chloride ions. The TPPE ligands form a two-dimensional (2D) network with an AB stacking arrangement, as previously reported by Chen et al.^34^

MOF **3** crystallizes in the monoclinic *P*2/*n* space group (Figures 1A and S2). The asymmetric unit contains a seven-coordinated Cd ion bound to two carboxylates, two pyridine units, and one water molecule. **3** features a 2D Cd-TPPE network similar to that in **2**, yet, the 2D networks are further locked by interlayer TCPE linkers to form a three-dimensional (3D) framework.

MOF **4** adopts a 3D metal–ligand framework similar to that of **3** with a calculated pore size of 1.3 nm (Figures S3 and S4). A key distinction between **3** and **4** lies in their metal coordination environments: in **4**, the six-coordinated Zn ion is in its most stable configuration, whereas in **3**, the seven-coordinated Cd ion is more dynamic due to the presence of a coordinated water molecule. Coordination solvent molecules in MOFs are known to be dynamic and readily removable, often creating unsaturated coordination sites that facilitate stronger interactions with guest molecules.^35−37^ The more flexible coordination environment in **3**, therefore, suggests superior sensitivity in practical applications.

As a result, compound **3** was selected as the representative MOF for pesticide sensing experiments, with **1**, **2**, and **4** serving as comparisons.

### Porosity and Fluorescence Properties

A comparison of the single-crystal structures of the as-synthesized MOFs highlights the importance of dual AIE linkers in pore modulation. Due to the small size of the TCPE ligand and the 3D interpenetrated topology resulting from chelating carboxylate groups, MOF **1** has an extremely small pore size of 0.2 nm (Figure S4). In contrast, MOF **2**, a two-dimensional (2D) structure, features a 1.5 nm window formed by the large TPPE ligand within its single-layer framework. However, its overall porosity is likely diminished by interlayer shifting, a common characteristic of 2D MOFs.^38,39^ The dual-linker design in MOFs **3** and **4** ensures robust porosity, as the TPPE contributes large 2D windows while the TCPE provides interlayer chelating interactions to stabilize the structure. Consequently, MOFs **3** and **4** retain larger pore sizes of 1.2 and 1.3 nm, respectively, as calculated from their single-crystal structures.

N_2_ adsorption analysis further validates the significantly higher porosity of MOFs **3** and **4** compared to MOFs **1** and **2**. The Brunauer–Emmett–Teller (BET) surface areas of **1**, **2**, **3**, and **4** are 5.5, 5.8, 220.3, and 363.4 m^2^·g^–1^ respectively (Figures 1B,C and S5). MOFs **1** and **2** exhibit negligible pore sizes, consistent with their structural properties. In contrast, MOFs **3** and **4** have measured pore sizes of 1.3 and 1.4 nm, closely aligning with the calculated values, confirming that the dual AIE linker strategy effectively enhances porosity as designed.

The second critical advantage of the dual AIE strategy—enhanced luminescence—is demonstrated through fluorescence characterization of the ligand monomers and their corresponding MOFs. First, the solid-state AIE effect in TCPE and TPPE was analyzed. As shown in Figure 1D, TPPE exhibits red-shifted excitation and emission spectra compared to TCPE, attributable to its larger conjugated π-system. Notably, the emission of TCPE overlaps with the excitation of TPPE, facilitating interligand energy transfer in dual-AIE MOFs.

To further explore the AIE effect, we compared the fluorescence of ligand monomers with that of their corresponding MOFs. The AIE effect arises from restricted aromatic ring rotation when ligands are tightly packed within the MOF structure.^40,41^ As shown in Figure 1E, TCPE, TPPE, and their mixture in dichloromethane (DCM) exhibit minimal fluorescence under 365 nm UV light. In contrast, MOFs **1**, **2**, and **3** exhibit intense blue or yellow fluorescence. The emission spectra of the MOFs (Figure 1F) reveal that MOFs **2** and **3** both feature emission peaks around 510 nm, similar to that of solid TPPE, suggesting energy transfer from TCPE to TPPE in MOF **3**. Furthermore, fluorescence lifetime measurements (Figure 1G) show that the dual-ligand MOF **3** has a longer fluorescence lifetime than either the ligand solids or the single-ligand MOFs, further confirming interligand energy transfer in MOF **3**.

Additionally, MOF **3** exhibits significantly stronger fluorescence intensity than MOFs **1** and **2**, demonstrating superior photoluminescence efficiency. The photoluminescence quantum yields (PLQYs) of MOFs **1**–**4** were determined to be 10.0, 51.5, 92.6, and 46.6%, respectively. The three-dimensional fluorescence spectrum of MOF **3** (Figure S6) shows that its strongest fluorescence emission occurs when excited at wavelengths between 365 and 380 nm, highlighting its suitability for practical applications under widely used 365 nm UV light.

### Pesticide Sensing Performance

To evaluate the sensing performance of MOF **3**, we tested its ability to detect 2,6-dichloro-4-nitroaniline (DCN), a widely used pesticide in fruit and vegetable cultivation. Excessive DCN residues pose significant health risks to humans.^42^ Conventional detection methods, such as mass spectrometry (MS) and high-performance liquid chromatography (HPLC), offer high sensitivity and accuracy but require complex sample preparation, extended processing times, and expensive, nonportable equipment.^43−46^ In contrast, MOF-based sensors present a promising alternative for on-site and rapid detection due to their ease of use, quick response times, and cost-effectiveness. However, existing MOF sensors often fall short in sensitivity, selectivity, anti-interference capability, and quantitativeness required to detect DCN in water at concentrations just above the regulatory residue limit (5–10 ppm).^42^ To date, no MOF-based sensor has been successfully demonstrated for practical field applications, such as detecting DCN contamination on fruits or in soil.

The structure of MOF **3** suggests it could achieve superior performance in DCN sensing for several reasons: (i) Its pore size is well-matched to the size of DCN molecules. (ii) It exhibits a high fluorescence quantum yield. (iii) The rotational restriction of AIE units in MOF **3** could be enhanced by interaction with the benzyl group of DCN. (iv) The coordination environment of MOF **3** may change upon interaction with DCN, further amplifying the sensing response.

**Figure 2:**
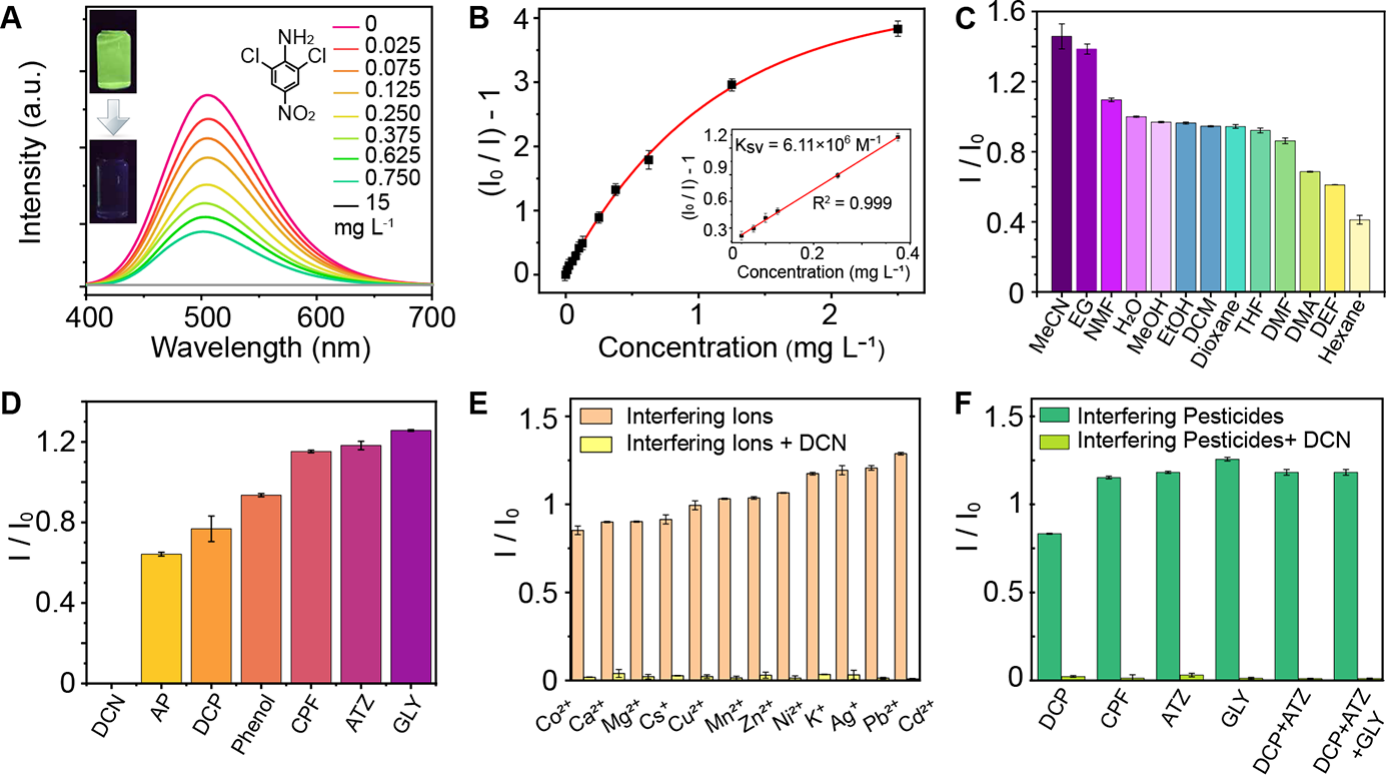
Dual-AIE MOF exhibits excellent visibility, sensitivity, stability, selectivity, and anti-interference capabilities for DCN detection. (A) Fluorescence quenching response of **3** to DCN. Inset shows the photos of **3** before and after exposure to 15 mg·L^–1^ DCN. (B) Fluorescence intensity as a function of varying DCN concentrations. Inset shows the linear fit for low DCN concentrations. (C) Fluorescence stability of **3** in various solvents. (D) Selectivity of **3** toward different pesticides: 2,6-dichloro-4-nitroaniline (DCN), 4-aminophenol (AP), 2,4-dichlorophenol (DCP), chlorpyrifos (CPF), atrazine (ATZ), and glyphosate (GLY). (E) Anti-interference performance of **3** against various metal ions. (F) Anti-interference performance of **3** against other pesticides.

The titration analysis (Figure 2A) shows that the fluorescence of MOF **3** decreases progressively with increasing concentrations of DCN (<1 mg·L^–1^), and is nearly completely quenched at 15 mg·L^–1^. The quenching efficiency follows the Stern–Volmer (SV) equation, *I*_0_/*I* = 1 + *K*_sv_·[M]. As shown in Figure 2B, the SV plot remains linear at DCN concentrations below 0.4 mg·L^–1^, yielding a *K*_sv_ value of 6.11 μM^–1^. The limit of detection (LOD) for DCN was determined to be 123 ppb based on the 3σ/*K*_sv_ method, representing the best-reported value for DCN detection using fluorescent sensors. In high-performance MOFs with a low detection limit, the active sites are easily saturated by the analyte due to the strong interactions, thus limiting the absolute concentration range of linear fluorescence response (25 to 375 ppb for MOF **3**, as shown in Figure 2B inset). Importantly, the narrow concentration range of linear response does not hinder practical quantitative detection. The wide concentration range for qualitative analysis and the known concentration range for quantitative analysis allow for straightforward dilution of high-concentration samples to bring them within the linear response range—an approach commonly used in other highly sensitive detection methods like HPLC and MS.

Table 1 summarizes representative methods for DCN detection, ranked by their LOD values. While techniques like HPLC and GC-MS offer exceptional sensitivity, they require intricate sample preparation and prohibitively expensive, nonportable instrumentation. Fluorescence- or colorimetry-based sensors, on the other hand, simplify sample preparation and eliminate the need for costly equipment. However, many of these sensors fail to meet regulatory limits for DCN residues in food (5–10 ppm).^42^ Notably, among sensors with LODs below permissible residue levels, only fluorescent MOFs have demonstrated required sensitivity, and MOF **3** stands out as the only one proven effective in water, a critical medium for real-world applications. Moreover, MOF **3** exhibits the lowest LOD and the fastest response time, achieving detection within 1 s (Figure S7). In subsequent sections, we demonstrate MOF **3**’s capacity for rapid, on-site DCN detection on fruits and soil, as well as its quantitative analysis capabilities using computer vision, underscoring its superior performance compared to other reported sensors.

**Table 1:** Summarization of Representative Methods for Sensing DCN

compound	material type	sensing method	LOD^a^	medium^b^	response time	demo.^c^	quant.^d^	reference
Cd–Sm nanocluster	nanoparticle	fluorescence	196.7 ppm	MeCN	N/A	no	no	(47)
TBAOH	ionic liquid	colorimetry	138 ppm	DMSO	3 min	yes	no	(48)
nonconjugated polymer dots	polymer	fluorescence	98.9 ppm	water	N/A	no	no	(49)
Tb_4_(BTDI)_3_(H_2_O)_4_	MOF	fluorescence	91.81 ppm	EtOH	N/A	no	no	(50)
UCNPs-HEP	nanoparticle	fluorescence	84.8 ppm	water	N/A	no	no	(51)
TfaTta–MB	COF	fluorescence	26.5 ppm	N/A	N/A	yes	no	(52)
Cd(tbia)· H_2_O	MOF	fluorescence	18.63 ppm	water	2 min	no	no	(46)
Eu_2_(dtztp)(OH)_2_(DMF)(H_2_O)_2.5_	MOF	fluorescence	5.28 ppm	water	1 min	no	no	(54)
regulatory limit			5–10 ppm					(42)
[H_3_O][Zn_2_L_1_(H_2_O)]	MOF	fluorescence	2.93 ppm	NMP	24 h aging	no	no	(55)
Zn_2_(L_2_)_2_(TPA)	MOF	fluorescence	390 ppb	MeOH	N/A	no	no	(56)
Mg_2_(APDA)_2_(H_2_O)_3_	MOF	fluorescence	150 ppb	DMF	N/A	no	no	(57)
Cd_3_(CBCD)_2_(DMA)_4_)(H_2_O)	MOF	fluorescence	145 ppb	DMA	N/A	no	no	(58)
Zn_2_(bpdc)_2_(BPyTPE)	MOF	fluorescence	130 ppb	DCM	N/A	no	no	(42)
**3**	MOF	fluorescence	123 ppb	water	1 s	yes	yes	this work
		HPLC	20 ppb	water	7.1 min^e^	yes	yes	(59)
		GC-MS	2 ppb	water	12.7 min^f^	yes	yes	(60)

a Ranked by the limit of detection from high to low, separated by the regulatory residue limit in food.

b “Medium” refers to the solutions used to disperse MOFs during DCN detection.

c “Demo.” indicates whether practical applications, such as detection on contaminated soil, beverages, or crops, have been demonstrated.

d “Quant.” specifies whether the capability for quantitative sensing has been established.

e Sample pretreatment is required prior to HPLC testing; the pretreatment time is not included in the reported response time.

f Sample pretreatment is required prior to GC-MS testing; the pretreatment time is not included in the reported response time.

Beyond sensitivity, we systematically evaluated the stability, selectivity, and anti-interference properties of MOF **3** for DCN sensing. As shown in Figure 2C, MOF **3** retains strong fluorescence in a variety of solvents, comparable to its performance in water, demonstrating excellent solvent stability.

Figure 2D highlights the high selectivity of MOF **3**. Its fluorescence is efficiently quenched by DCN but remains largely unaffected by other pesticides, such as chlorpyrifos (CPF), atrazine (ATZ), and glyphosate (GLY), or by structurally similar compounds like 4-aminophenol (AP) and 2,4-dichlorophenol (DCP). Additionally, anti-interference tests (Figure 2E,F) confirm that DCN efficiently quenches the fluorescence of MOF **3**, while additives, including various metal ions and pesticides, have no significant effect. MOF **3** also exhibits strong resistance to pH changes and interference from various cations (Figure S8).

Comparative studies with MOFs **1**, **2**, and **4** revealed significantly weaker responses to DCN compared to MOF **3** (Figure S9). The low sensitivity of MOFs **1** and **2** is attributed to their limited porosity, which restricts the absorption and interaction with DCN molecules. In contrast, the weak response of MOF **4** is likely due to the saturated coordination environment of its Zn center, which fails to provide available binding sites. MOF **3**, however, combines a porous structure with a dynamic coordination environment, enabling its exceptional performance in DCN detection.

### Investigations on the Sensing Mechanism

**Figure 3:**
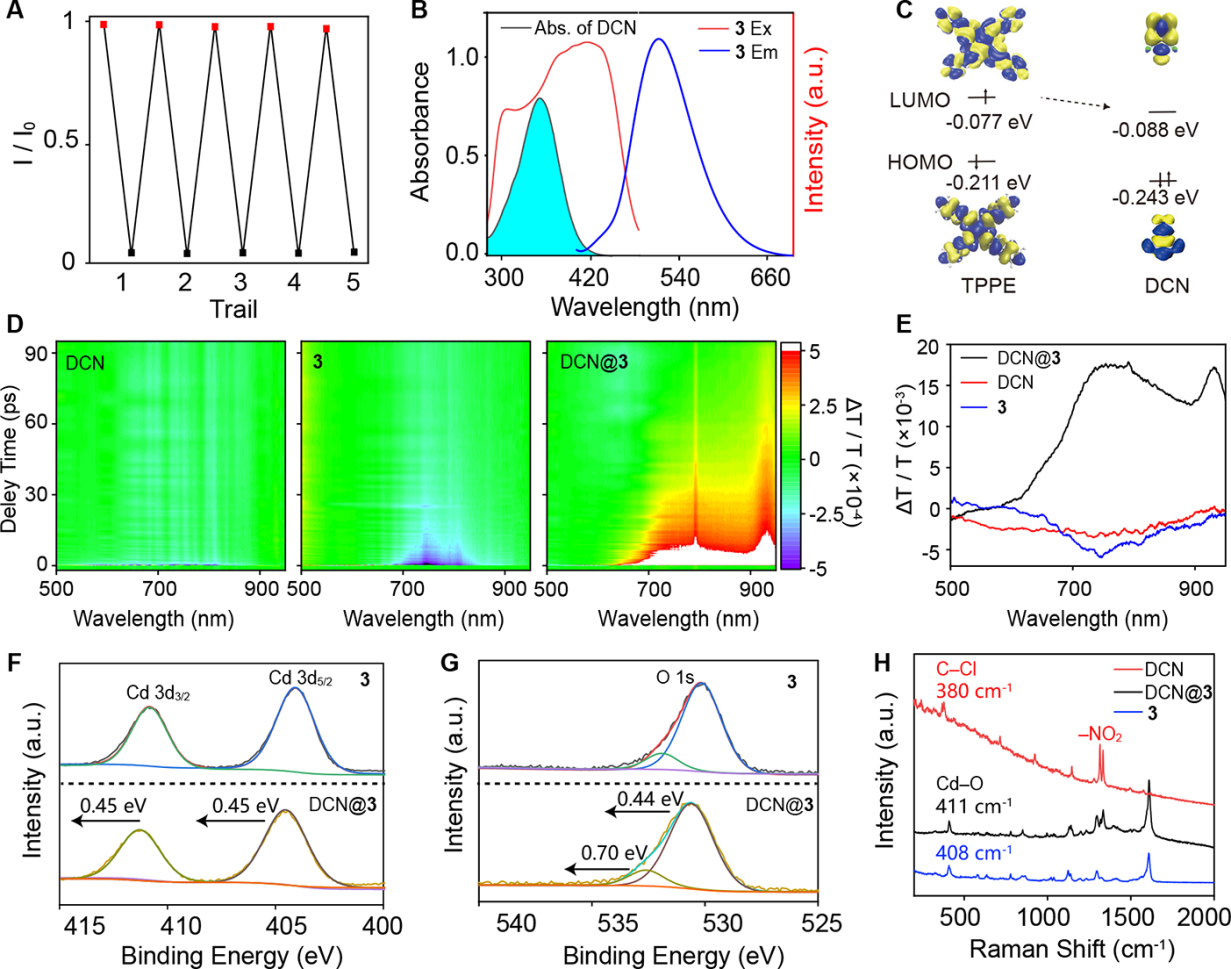
Investigating the fluorescence sensing mechanism. (A) Reproducibility of DCN sensing after multiple experimental cycles. After each cycle, **3** was regenerated by ethanol washing. (B) Comparison of the UV–vis spectrum of DCN and the fluorescence spectrum of **3**. (C) Diagram of the calculated LUMO and HOMO energy levels of TPPE and DCN. (D) Time-resolved transient absorption spectra of DCN, **3**, and DCN@**3**. (E) Transient absorption spectra at 0.5 ps. (F, G) X-ray photoelectron spectra of MOF **3** and DCN@**3** in the regions of the Cd 3d and O 1s peaks. (H) Raman spectra of DCN, **3**, and DCN@**3**.

Common mechanisms for fluorescence quenching include structural degradation, Förster resonance energy transfer (FRET), competitive absorption, and photoinduced electron transfer (PET).^61,62^ Recycling experiments confirmed that the fluorescence quenching observed in MOF **3** is not due to structural collapse. After five cycles of DCN sensing and ethanol washing, the fluorescence intensity of **3** remained largely unchanged, with quenching efficiency remaining nearly complete (Figure 3A). Furthermore, the powder X-ray diffraction (PXRD) patterns of **3** before and after DCN sensing were nearly identical and matched closely with the simulated pattern (Figure S10), verifying structural integrity.

As shown in Figure 3B, the emission spectrum of MOF **3** spans from 400 to 800 nm, while the UV–vis absorption of DCN is limited to 280–430 nm, ruling out the possibility of FRET from **3** to DCN.

Although Figure 3B also suggests potential competitive absorption due to the overlap between the excitation wavelength of MOF **3** and the absorption spectrum of DCN, this mechanism alone does not explain the efficient quenching. In a mixed solution, a concentration of 15 mg L^–1^ DCN completely quenches the fluorescence of MOF **3** (Figure 2A), even though 17% of the excitation light at 365 nm can still pass through the DCN solution (corresponding to an absorbance of 0.78). Control experiments (Figure S11) further confirm this; even a 10-fold concentrated DCN solution, when placed separately in front of the light source, fails to quench the fluorescence of MOF **3** effectively. These results indicate that while competitive absorption exists, it is not the primary cause of the observed quenching.

We attribute the efficient fluorescence quenching to photoinduced energy transfer (PET), supported by transient absorption spectroscopy and theoretical calculations. Density functional theory (DFT) calculations (Figure 3C) indicate that the lowest unoccupied molecular orbital (LUMO) of TPPE in MOF **3** is aligned with the LUMO of DCN, facilitating PET. Transient absorption spectra (Figure 3D,E) reveal that both DCN and MOF **3** exhibit ground-state bleaching under 400 nm laser excitation when analyzed individually. In contrast, DCN@**3** exhibits strong excited-state absorption, confirming the occurrence of PET from MOF **3** to DCN.

Beyond fluorescence quenching, the interaction between MOF **3** and DCN is further evidenced by Brunauer–Emmett–Teller (BET) analysis, X-ray photoelectron spectroscopy (XPS), and Raman spectroscopy. BET analysis (Figure S12) shows that DCN enters the pores of MOF **3**, reducing the surface area from 220.3 to 3.5 cm^3^ g^–1^. XPS data (Figure 3F,G) reveal that DCN absorption induces positive shifts in the binding energies of Cd 3d_5/2_ (from 404.15 to 404.60 eV) and Cd 3d_3/2_ (from 410.90 to 411.35 eV), as well as O 1s peaks (from 531.90 to 532.60 eV and from 530.16 to 530.60 eV), indicating strengthened Cd–O bonding after DCN adsorption. Raman spectroscopy (Figure 3H) further supports this, showing that the Cd–O vibrational peak shifts to higher frequencies upon DCN absorption. Additionally, the Raman spectra reveal a relative intensity change in two vibrational peaks of the nitro group and significant suppression of the C–Cl vibrational peaks in DCN@**3**.^63^

Combining these observations with the unsaturated coordination of Cd centers in MOF **3**, we hypothesize that the interaction between MOF **3** and DCN involves alteration of the Cd coordination environment via synergistic interactions with the nitro and chloro groups of DCN. This mechanism explains the exceptional sensing selectivity of MOF **3** for DCN while being significantly less responsive to other pesticides, such as AP and DCP. It also accounts for the lower sensing sensitivity of MOF **4**, which features saturated coordination at the Zn centers.

### Quantitative Visual Sensing in Practical Application

To demonstrate the practical utility of MOF **3**, we applied it for the detection of DCN residues on *Clausena lansium* (Lour.) Skeels fruits. In agricultural practices, DCN is typically applied by spraying a 0.3 M solution onto crops.^64^ To simulate this process, nine clean *Clausena lansium* fruits were selected, and four of them were briefly dipped into a 0.3 M DCN solution. All nine fruits were subsequently coated with a suspension of MOF **3**, air-dried, and arranged in a 3 × 3 grid for visual detection and imaging. The entire coating and drying procedure was completed within minutes, demonstrating the rapidity and efficiency of this method for pesticide residue detection.

**Figure 4:**
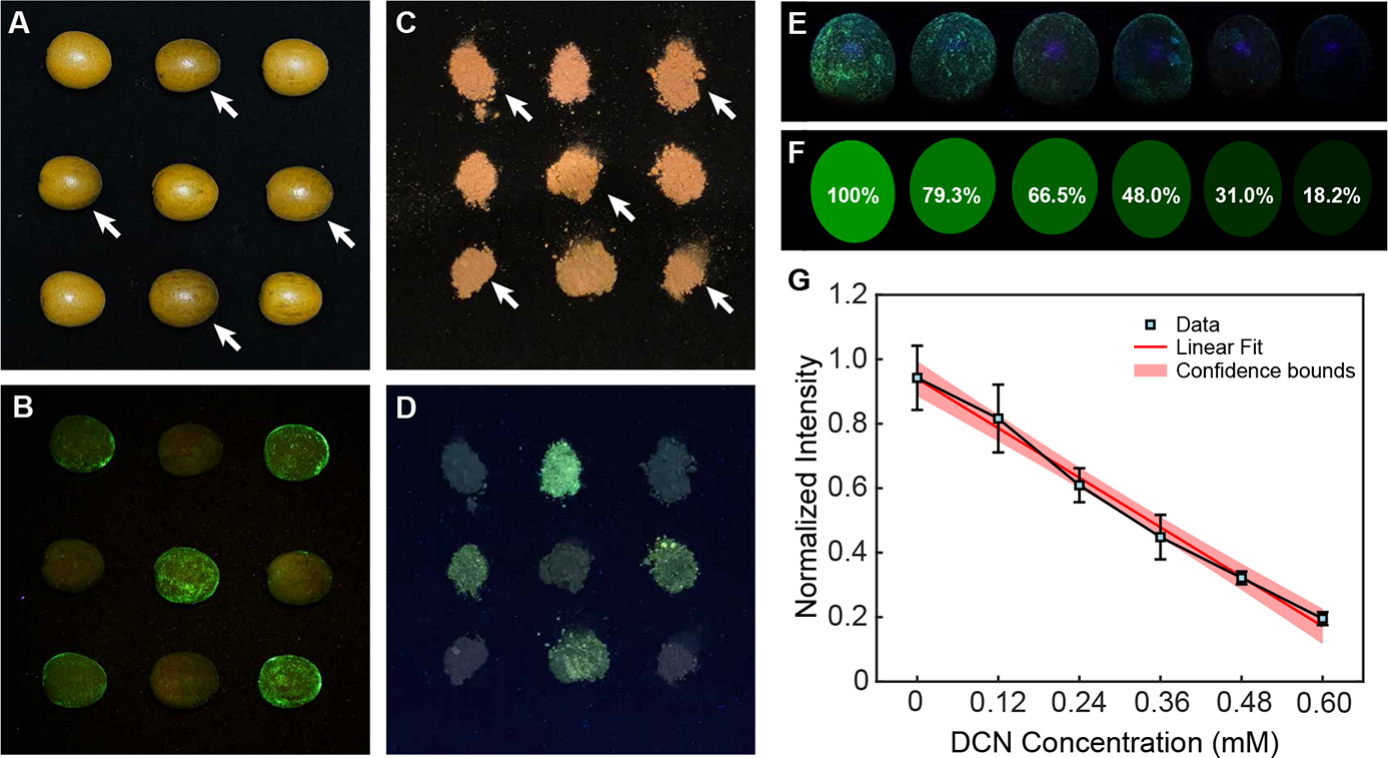
Direct visual detection and quantitative analysis of DCN in practical applications. (A) Photo of nine *Clausena lansium* fruits under daylight, with four fruits contaminated by DCN (indicated by arrows). (B) Photo of the same fruits, sprayed with **3** and illuminated under 365 nm UV light. (C) Photo of nine piles of soil under daylight, with five piles contaminated by DCN (indicated by arrows). (D) Photo of the same soil, sprayed with **3** and illuminated under 365 nm UV light. (E) Photos of fruits after immersion in solutions with varying DCN concentrations (0 to 0.6 mM), sprayed with **3**, and illuminated under 365 nm UV light. (F) Computer vision analysis of panel (C), showing the recognized fruit shapes and normalized fluorescence intensities. (G) Linear fit of the recognized fluorescence intensity as a function of DCN concentration.

As shown in Figure 4A, no visible difference was observed between the clean and DCN-treated fruits under daylight. However, when illuminated under 365 nm UV light (19 mW·cm^–2^), the clean fruits emitted bright green fluorescence visible to the naked eye (Figure 4B). In contrast, the fluorescence from the DCN-treated fruits was entirely quenched. This striking difference highlights the exceptional performance of MOF **3** for visual pesticide detection in real-world fruit samples.

To further explore the practicality of the sensor in more complex matrices, we conducted similar experiments using soil samples. Three soil samples were collected, washed with water, and divided into three portions each. Five of these portions were treated with DCN solutions. All nine samples were coated with the MOF suspension, air-dried, and arranged in a 3 × 3 grid for visual detection and imaging.

As illustrated in Figure 4C, no noticeable differences were observed between untreated and DCN-treated soil samples under daylight. However, upon excitation with 365 nm UV light, the untreated samples exhibited bright green fluorescence, whereas the fluorescence of the DCN-treated samples was completely quenched (Figure 4D). These results demonstrate the exceptional capability of MOF **3** for DCN sensing in diverse soil types, underscoring its potential applicability in complex environmental settings.

To enable quantitative detection, we utilized computer vision analysis to evaluate DCN residues at varying concentrations. Elliptical kernels were applied to UV-illuminated images to identify the fruit shapes, and fluorescence intensity was quantified as the normalized brightness of the green channel relative to a clean fruit (Figure 4E,F).

As the DCN concentration increased from 0 to 0.6 mM, the fluorescence intensity decreased from 100 to 18.2%. Measurements were performed on three fruits for each concentration, and the resulting fluorescence intensities, along with their standard errors, were plotted in Figure 4G. The observed linear relationship between fluorescence intensity and DCN concentration, combined with the small standard errors, confirms the reliability and accuracy of this simple, rapid, and user-friendly method for quantifying pesticide residues in real-world applications.

Although MOF **3** exhibits excellent stability and strong resistance to interference, it decomposes under strongly basic conditions (pH > 13). Therefore, the pH of samples should be measured and adjusted to ensure functional sensing in practical applications. Additionally, in quantitative analyses, high-quality UV light with stable and uniform illumination is essential to achieve reliable and reproducible data for accurate quantification.

## Conclusions

In summary, we have designed and synthesized a dual-AIE MOF along with its analogous structures, showcasing exceptional pesticide detection performance enabled by the synergy of two AIE linkers. The complementary coordination groups of the linkers form a robust 3D framework with an optimized pore structure and a dynamic coordination environment. The energy transfer between the two AIE linkers results in an impressive photoluminescent quantum yield of 92.6%. Capitalizing on these features, the dual-AIE MOF achieves unparalleled sensitivity for detecting DCN, exhibiting remarkable selectivity, rapid response, quantitativeness, recyclability, and resistance to interference. Transient absorption spectroscopy and theoretical calculations attribute the highly efficient fluorescence quenching to photoinduced energy transfer. Moreover, we developed a practical approach for quantitative pesticide residue detection by applying the dual-AIE MOF to real fruit and soil samples and analyzing UV-illuminated images using computer vision. This work establishes a reliable, instantaneous, and quantitative visual detection strategy using luminescent MOFs (LMOFs) for real-world applications. It provides valuable insights into the synthesis and application of MOFs with synergistic AIE units, while also demonstrating the potential of integrating high-performance visual sensors with computer vision technology for on-site, practical detection.

## Supporting Information Available

Synthetic details of all ligands, MOFs and sensing experiments, single crystal structure and characterization of MOFs, and other supplement sensing experiments (PDF)

## Accession Codes

X-ray crystallographic data for **1**, **3**, **4**. Detailed crystallographic data can be obtained free of charge from The Cambridge Crystallographic Data Centre via www.ccdc.cam.ac.uk/datarequest/cif (CCDC: 2392414–2392416).
