# Supplementary material for: Synergistic Aggregation-Induced Emissive Linkers in Metal–Organic Frameworks for Ultrasensitive and Quantitative Visual Sensing
Source: JACS Au. 2025 Mar 17;5(4):1875–83. doi: 10.1021/jacsau.5c00092 (PMC12042037; doi:10.1021/jacsau.5c00092)
Supplement: Supplementary file 1 — au5c00092_si_001.pdf [file au5c00092_si_001.pdf]

Supplementary information for

Synergistic Aggregation-Induced Emissive Linkers in Metal-Organic Frameworks for Ultrasensitive and Quantitative Visual Sensing

Yansong Jiang[1,†], Wenxin Chang[1,†], Zhihao Li[1,2,†], Xiang Zhou[1], Panjing Zhang[1], Xuehai Huang[1], Xinyi Pan[1], Zhenda He[1], Yu Wang[1,3,4*], and Zhongqun Tian[2]

Affiliations:

[1]South China Advanced Institute for Soft Matter Science and Technology, School of Emergent Soft Matter, South China University of Technology, Guangzhou 510640, China.

[2]State Key Laboratory of Physical Chemistry of Solid Surfaces and College of Chemistry and Chemical Engineering, Xiamen University, Xiamen, 361005, China.

[3]Center for Electron Microscopy, South China University of Technology, Guangzhou 510640, China.

[4]Guangdong Provincial Key Laboratory of Functional and Intelligent Hybrid Materials and Devices, South China University of Technology, Guangzhou 510640, China.

[†]Contributed equally to this work.

[*]To whom correspondence should be addressed; E-mail: roywangyu@scut.edu.cn

This PDF file includes:

1 Supplementary Figures **2**

S1. Single-crystal structure of **1** 2
S2. Single-crystal structure of **3** 3
S3. Single-crystal structure of **4** 3
S4. The calculated van der Waals radii 4
S5. The N_2 adsorption isotherm of **4** 4
S6. 3D fluorescence spectrum of **3** 5
S7. Response time of sensing DCN with **3** 5
S8. The anti-interference analysis of **3** 5
S9. The fluorescent sensing performance of **1-4** for different pesticides 7
S10. PXRD patterns of MOF **3** before and after DCN sensing 7
S11. Fluorescence change when mixed with or blocked by DCN solutions 7
S12. N_2 adsorption isotherm of **3** and DCN@**3** 7

2 Supplementary Methods **9**

S1. Synthesis and sample preparation 9
S2. Determination of crystal structures 11
S3. Fluorescence sensing experiments 11
S4. Fluorescent lifetime measurement 12
S5. Femtosecond transient absorption measurement 12
S6. Raman measurement 12
S7. Theoretical calculations 13

1 Supplementary Figures

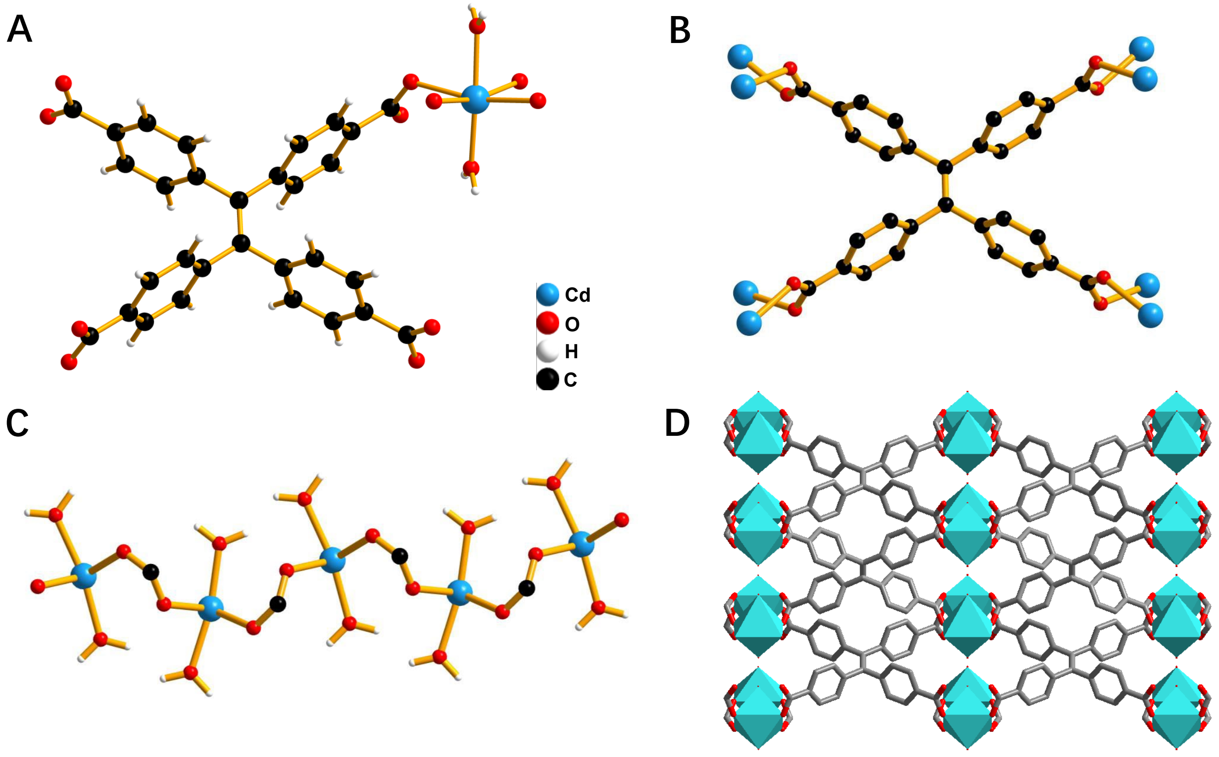

Fig. S1 | Single-crystal structure of 1. **A**, Coordination environment of Cd in **1**. **B**, Coordination mode of TCPE. **C**, One-dimensional chain in **1**. **D**, 3D framework of **1**.

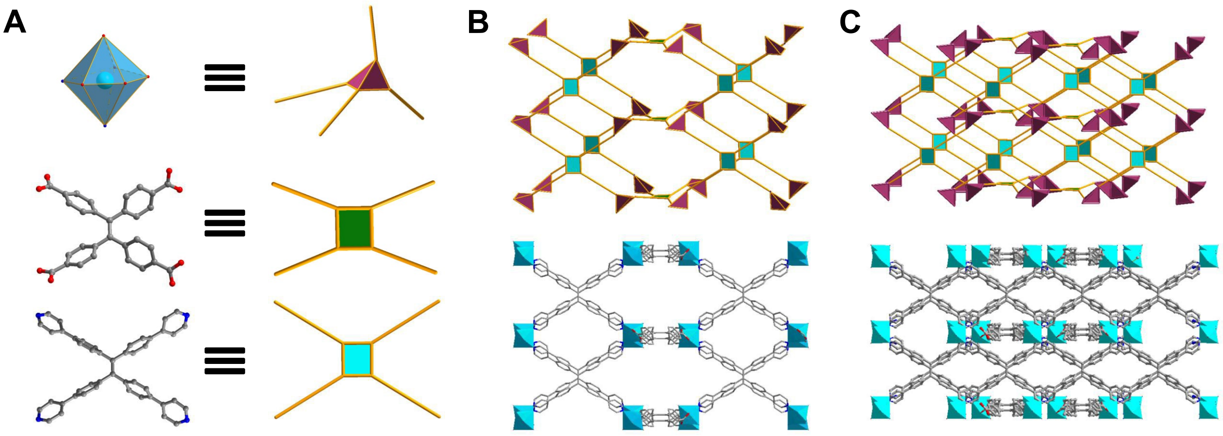

Fig. S2 | Single-crystal structure of 3. **A**, Building blocks. **B-C**, Framework topology.

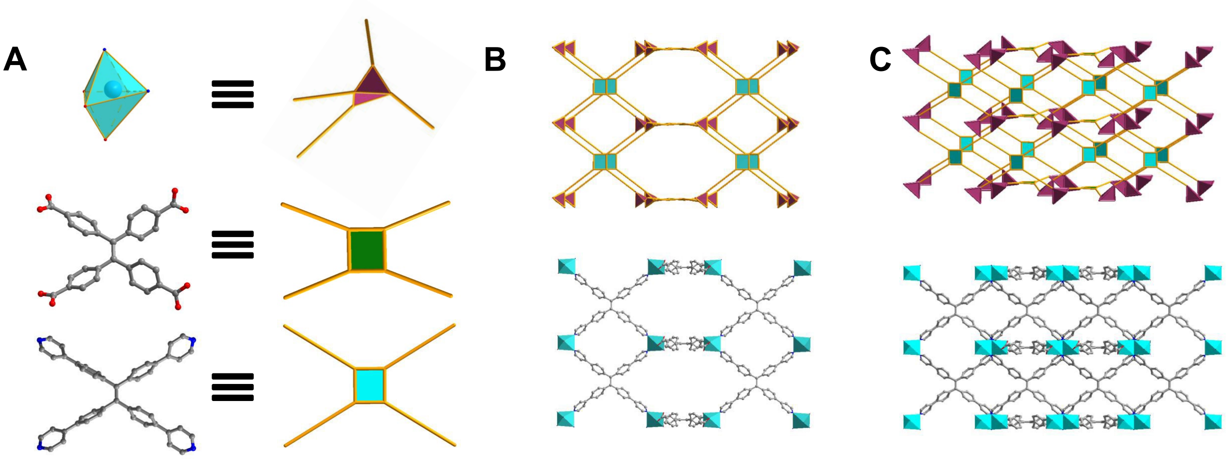

Fig. S3 | Single-crystal structure of 4. **A**, Building blocks. **B-C**, Framework topology.

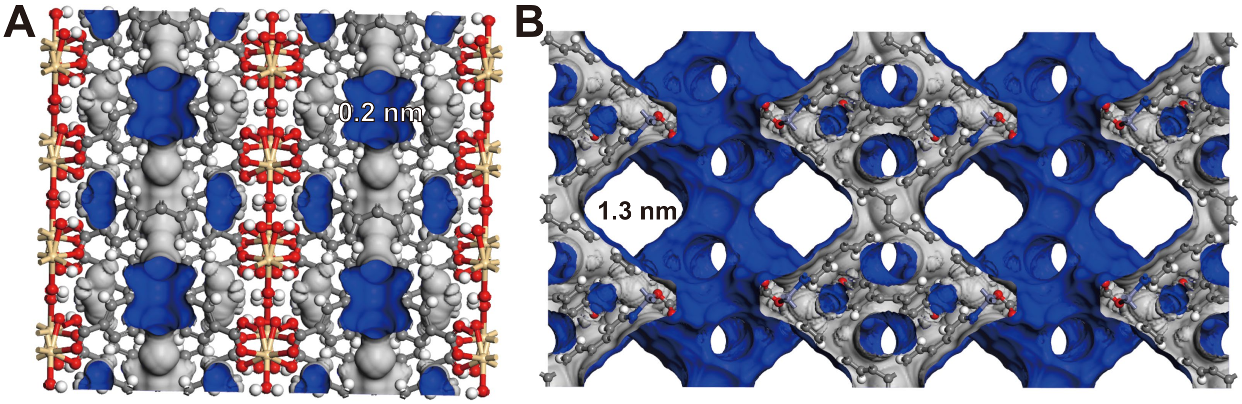

Fig. S4 | The van der Waals radii of pores in **1** (A) and **4** (B).

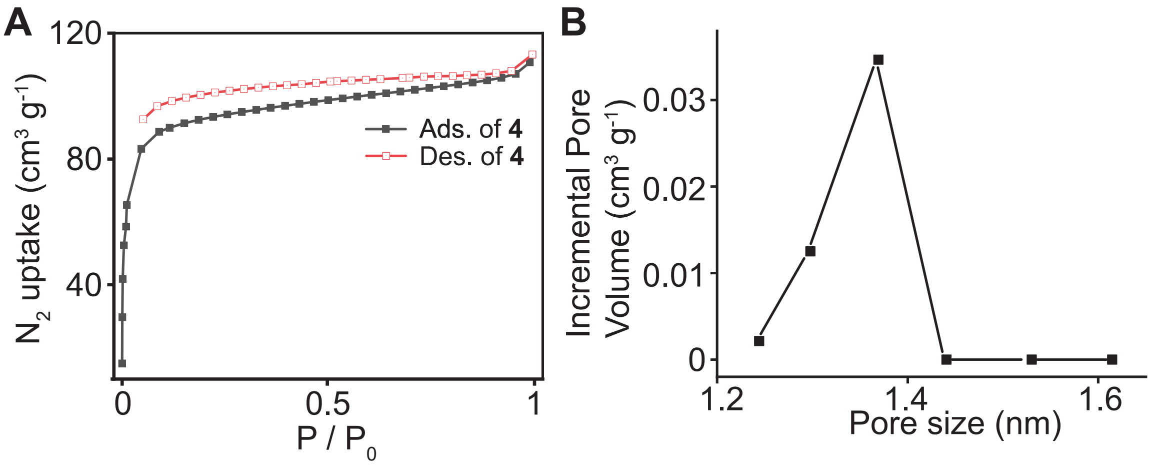

Fig. S5 | N_2 adsorption isotherm of **4**. **A**, The N_2 adsorption isotherm at 77 K; **B**, The pore size distribution of **4**.

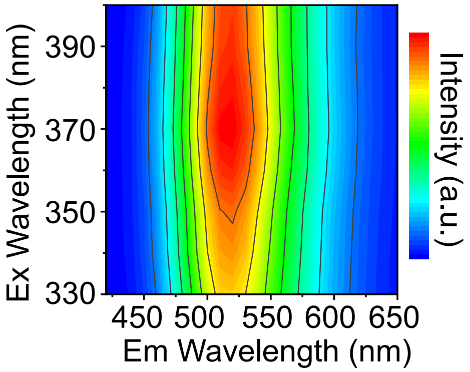

Fig. S6 | 3D fluorescence spectrum of **3**.

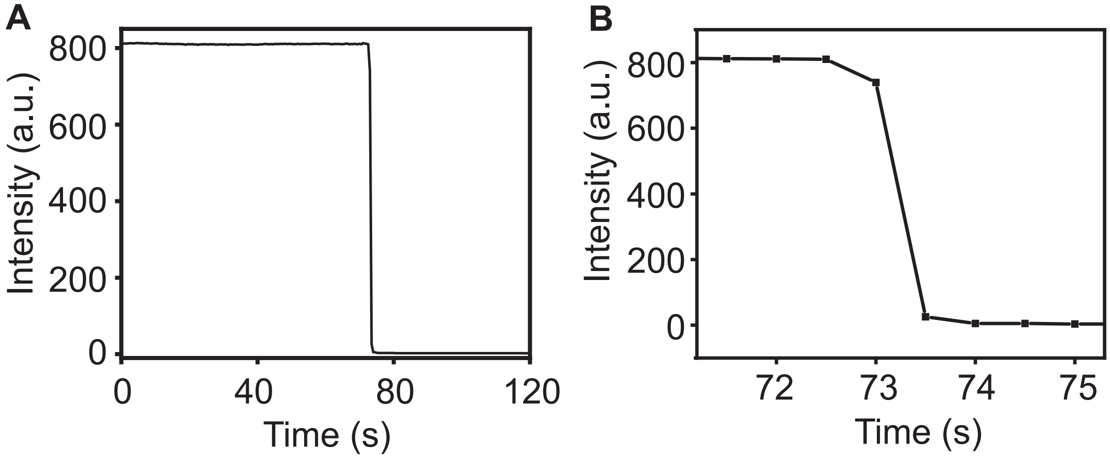

Fig. S7 | Response time of sensing DCN with **3**. **A**, Fluorescence intensity change of MOF **3** at 510 nm upon the addition of DCN (0.3 M). **B**, Magnified view highlighting the rapid fluorescence quenching.

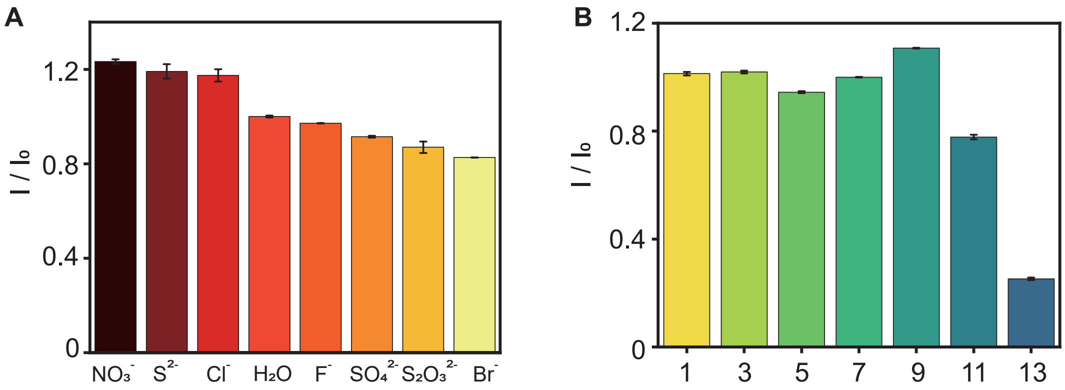

Fig. S8 | The anti-interference analysis of **3** against anions and pH. **A**, The anti-interference analysis of **3** against different anions at the same concentration of DCN (50 mg L^{-1}). **B**, The fluorescent intensity of **3** under different pH conditions.

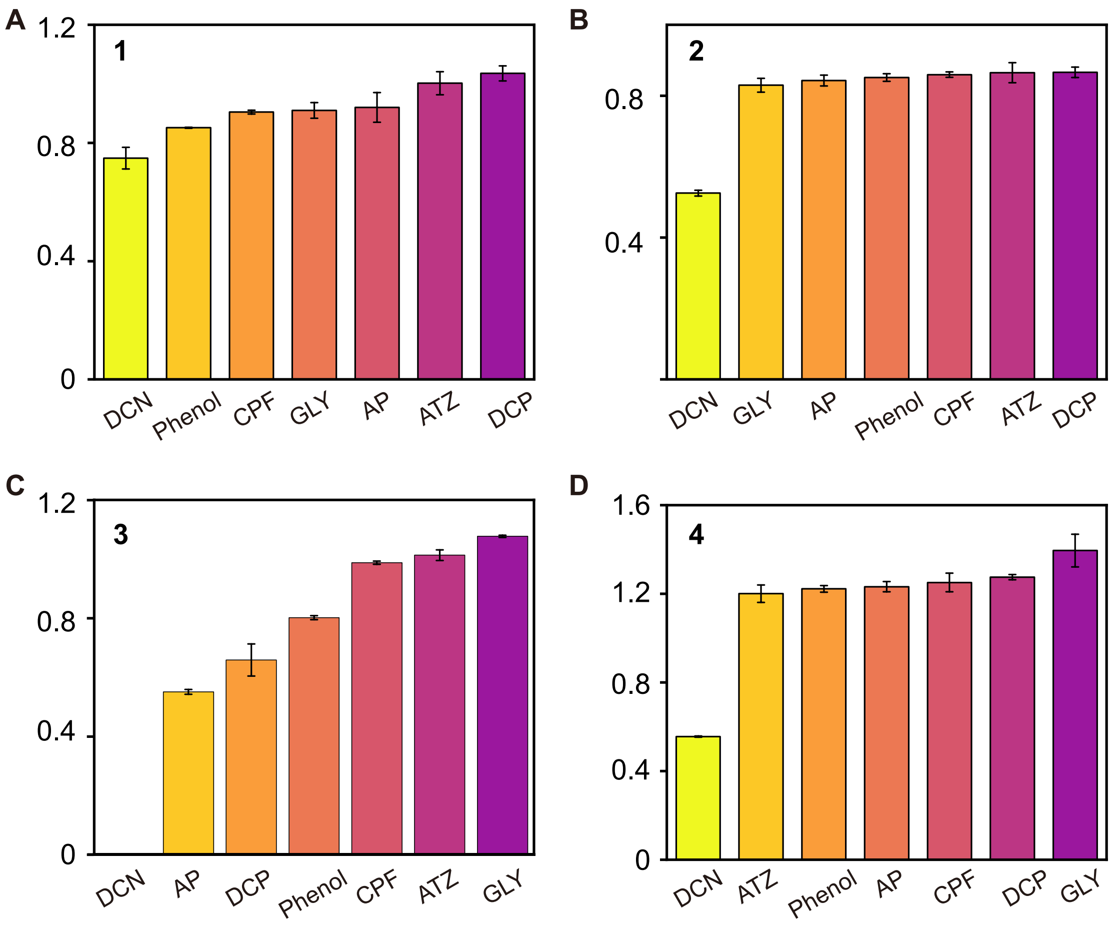

Fig. S9 | The fluorescent sensing performance of **1** (A), **2** (B), **3** (C), and **4** (D) for different pesticides.

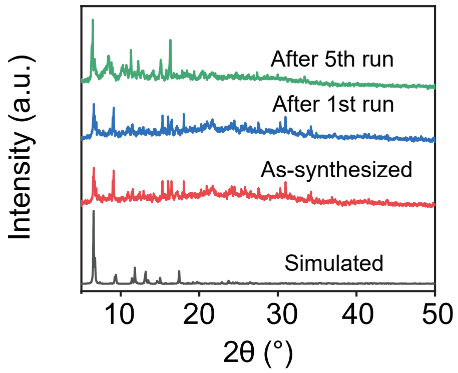

Fig. S10 | PXRD patterns of MOF **3** before and after DCN sensing.

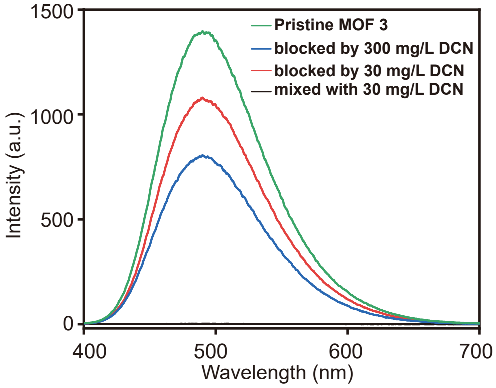

Fig. S11 | Fluorescence changes of MOF **3** when directly mixed with DCN or when blocked by isolated DCN solutions.

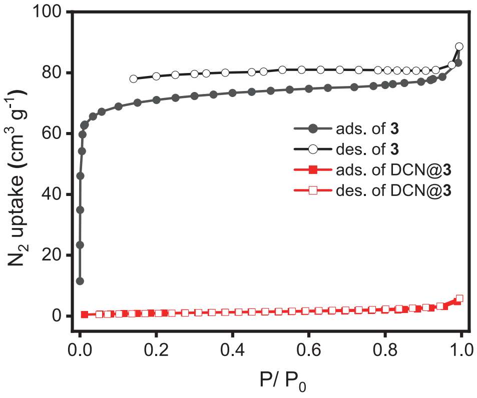

Fig. S12 | N_2 adsorption isotherm of **3** and DCN@**3**.

2 Supplementary Methods

S1. Synthesis and sample preparation

The syntheses of ligands were according to the reported procedure, with modifications.[1,2]

Synthesis of TPE-Br_4

TPE (3.32 g) was dissolved in 60 mL of dichloromethane (DCM), and Br_2 (7 mL) was added dropwise while maintaining an ice bath. The reaction mixture was stirred for 12 hours and then quenched with ethanol and sodium thiosulfate ($Na_2S_2O_3$). Sodium hydroxide (NaOH) solution was added to adjust the pH to 7. The organic phase was extracted with DCM three times, yielding a crude powder product. Yield: 98%. ^{1}H NMR (400 Hz, $CDCl_3$, δ): 7.26 (d, 8H), 6.84 (d, 8H).

Synthesis of TPE-CN_4

TPE-Br_4 (5.0 g) and CuCN (4.2 g) were dissolved in 20 mL of dry dimethylformamide (DMF), and the mixture was refluxed for two days under a nitrogen atmosphere. After cooling to 60 °C, $NH_3 \cdot H_2O$ (20 mL) and H_2O (50 mL) were added, followed by stirring for an additional 2 hours. The mixture was filtered, and the filtrate was extracted with DCM three times. The organic phase was dried over Na_2SO_4 and evaporated to yield a yellow product. Yield: 72%. ^{1}H NMR (400 Hz, $CDCl_3$, δ): 7.48 (t, 8H), 7.08 (d, 8H).

Synthesis of H_4TCPE

TPE-CN_4 (0.86 g) and KOH (2.24 g) were added to 30 mL of ethylene glycol and heated at 190 °C for three days. After cooling to room temperature, HCl (6 M) was added dropwise in an ice bath until the pH reached 2. The pale yellow powder product was collected and washed with water. Yield: 94%. ^{1}H NMR (400 Hz, DMSO-d_6, δ): 12.89 (s, 1H), 7.74 (d, 8H), 7.12 (d, 8H).

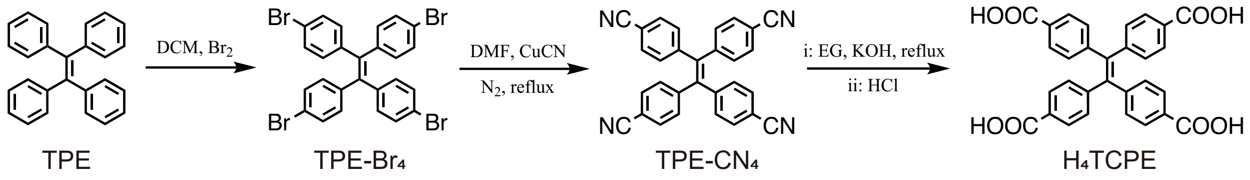

Scheme S1 | The synthetic route of H_4TCPE.

Synthesis of TPPE

TPE-Br_4 (1.00 g), pyridine-4-boronic acid (1.14 g),K_2CO_3 (2.76 g), and $Pd(PPh_3)_4$ (0.27 g) were combined in 60 mL of a toluene/ethanol/water mixture (4:1:1) and refluxed for 24 hours under a nitrogen atmosphere. The reaction mixture was extracted with DCM three times, followed by evaporation. The crude product was purified via column chromatography using DCM/methanol (10:1) as the eluent, resulting in a yellow-green powder. Yield: 83%. ^{1}H NMR (400 Hz, $CDCl_3$, ppm): 8.65 (d, 8H), 7.56-7.49 (m, 16H), 7.24 (d, 8H).

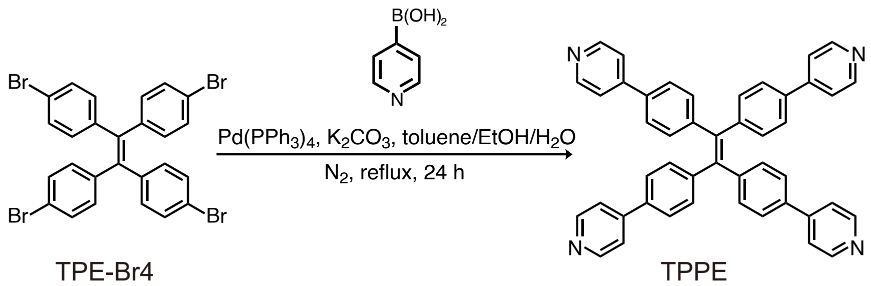

Scheme S2 | The synthetic route for TPPE.

Synthesis of 1

In a 25 mL Teflon-lined vessel, $Cd(NO_3)_2 \cdot 4H_2O$ (25 mg, 0.081 mmol) and H_4TCPE (6 mg, 0.012 mmol) were dissolved in 6 mL of an acetonitrile/water mixture (v/v = 2:1). The pH was adjusted with 0.2 mL of HNO_3. The vessel was sealed in an autoclave and heated at 160 °C for 24 hours. Upon cooling to room temperature, colorless prismatic crystals were obtained. Yield: 71% (based on $Cd(NO_3)_2 \cdot 4H_2O$). FT-IR (ATR, cm^{-1}): 3148 (m), 1650 (w), 1575 (s), 1499 (s), 1388 (s), 1297 (w), 1177 (m), 1135 (w), 1108 (w), 1014 (m), 966 (w), 879 (m), 838 (m), 765 (s), 713 (m), 647 (m), 571 (m), 505 (w), 482 (w).

Synthesis of 2

As reported before[3], $CdCl_2$ (22.8 mg, 0.10 mmol), TPPE (8 mg, 0.0125 mmol), DMF (6 mL), and 30 μL of nitric acid (2 mL of concentrated nitric acid in 6 mL of DMF) were placed in a 15 mL Teflon reactor and heated at 120 °C for 3 days.

Synthesis of 3

In a 20 mL flask, $Cd(NO_3)_2 \cdot 4H_2O$ (24.67 mg, 0.08 mmol), H_4TCPE (6.10 mg, 0.012 mmol), and TPPE (5.78 mg, 0.009 mmol) were dissolved in 4 mL of DMF. HBF_4 (0.1 mL, 48 wt.%) was subsequently added, and the mixture was heated at 105 °C for two days. After cooling to room temperature, light yellow crystals were collected by filtration, washed with DMF and ethanol, and air-dried. Yield: 65% (based on $Cd(NO_3)_2 \cdot 4H_2O$). FT-IR (ATR, cm^{-1}): 3342 (m), 1602 (s), 1578 (s), 1530 (s), 1374 (s), 1222 (w), 1177 (w), 1100 (w), 1066 (w), 1014 (m), 817 (w), 769 (s), 710

(s), 645 (m), 482 (w).

Synthesis of 4

In a 20 mL flask, $Zn(NO_3)_2 \cdot 6H_2O$ (17.85 mg, 0.06 mmol), H_4TCPE (6.10 mg, 0.012 mmol), and TPPE (5.78 mg, 0.009 mmol) were dissolved in 4 mL of DMF. Subsequently, 0.4 mL of HBF_4 (48 wt.%) was added, and the mixture was heated at 105 °C for two days. After cooling to room temperature, light yellow crystals were collected by filtration, washed with DMF and ethanol, and air-dried. Yield: 58%, based on $Zn(NO_3)_2{\cdot}6H_2O$. FT-IR (ATR, cm^{-1}): 3360 (m), 1595 (s), 1540 (m), 1488 (m), 1391 (s), 1229 (m), 1177 (m), 1108 (m), 1069 (w), 1042 (w), 813 (m), 765 (s), 713 (m), 644 (w), 557 (w), 496 (m), 436 (m).

S2. Determination of crystal structures

Single crystals of compounds **1**, **3**, and **4** with the appropriate size, shape, and transparency were selected under an optical microscope for single-crystal X-ray diffraction (XRD) analysis. Diffraction data were collected using a Rigaku XtaLAB Synergy-DW diffractometer with graphite-monochromated Mo-Kα radiation at 150 K. Crystal structures were solved by direct methods and refined using full-matrix least-squares on F^2 with OLEX2 and SHELXTL-2014 software. Hydrogen atoms were geometrically positioned and refined using a riding model, while non-hydrogen atoms were refined anisotropically. Residual electron density was addressed using a solvent mask program.[4,5] CCDC-2392414-2392416 contains the supplementary crystallographic data.

S3. Fluorescence sensing experiments

For fluorescence sensing experiments, 1.4 mg of the MOF powder was added to 2 mL of H_2O containing inorganic ions, organic solvents, or pesticide solutions (0.2 mL, 50 mg L^{-1}). The samples were subjected to ultrasonic treatment for several minutes before collecting fluorescence spectra.

For the anti-interference experiments, 1.4 mg of MOF powder was dispersed in 2 mL of H_2O containing 0.2 mL of DCN (50 mg L^{-1}) and interfering substances at the same concentration for fluorescence testing. All fluorescence spectra were recorded in triplicate, and the average values were used.

The recycling experiments followed the same procedure as the sensing experiments but reused MOF from previous runs. The DCN was removed from **3** by immersing it in EtOH for about 30 min for three times. After drying and dispersing, the fluorescence will be recovered.

For the practical DCN sensing experiments, *Clausena lansium* (Lour.) Skeels fruits were immersed in DCN solution (0.3 M), after which the DCN-treated samples were dipped in a 2 g L^{-1} MOF suspension. Following air drying, four MOF-coated, DCN-treated samples and five

MOF-coated, non-treated samples were arranged in a 3 × 3 array within a dark box, positioned 68 cm from a UV lamp (365 nm, 19 mW cm^{-2}).

For the quantified DCN sensing experiment, *Clausena lansium* (Lour.) Skeels fruits were immersed in DCN solutions at concentrations of 0.12, 0.24, 0.36, 0.48, and 0.60 mM. The DCN-treated samples were then coated with a 2 g L^{-1} MOF suspension, and fluorescence was recorded.

For the practical DCN sensing experiment in soil, the soil from Guangzhou international campus of South China University of Technology, Wushan campus of South China University of Technology, and by the lakeside are collected and washed with water three times. The dried soil are immersed in the DCN solution (0.3 M) and oven dried. Then, it DCN treated and non-treated soil are immersed with MOF **3** solution (2 g L^{-1}, ethanol solution) and dried for use. Four MOF-coated non-treated samples and five MOF-coated DCN-treated samples are arranged in a 3 × 3 array within a dark box, positioned 68 cm from a UV lamp (365 nm, 19 mW cm^{-2}).

Computer vision script was written in Python. Elliptical masks with different sizes and ellipses were applied on the UV-illuminated photo, and the optimization of the correlated images lead to the recognition of fruits. The mean intensity of the green channel in the recognized fruit areas were used for plotting Fig. 4G.

S4. Fluorescent lifetime measurement

Fluorescence lifetime measurements were conducted using an Edinburgh FLS1000 Photoluminescence Spectrometer equipped with a 375 nm pulsed laser.

S5. Femtosecond transient absorption measurement

Transient absorption measurements of MOF **3**, DCN, and DCN@MOF **3** were performed using the following setup. A femtosecond laser (PHAROS, Light Conversion) operating at a repetition rate of 100 kHz served as the light source for the Femto-TA100 spectrometer (Time-Tech Spectra Co., Ltd). The laser pulse duration was approximately 290 fs, with an output wavelength of 1030 nm. The fundamental laser beam was split into two components: one was directed to an optical parametric amplifier (OPA, Light Conversion) to generate a 400 nm pump beam for the Femto-TA100 system, while the other was focused onto a sapphire plate to produce supercontinuum white light, which served as the probe beam. A motorized delay stage was employed to adjust the time delay between the pump and probe beams. All measurements were carried out at room temperature under ambient atmospheric conditions.

S6. Raman measurement

Raman spectra of DCN, MOF **3**, and DCN@MOF **3** were recorded using a Renishaw inVia Qontor Raman confocal spectrometer with a 785 nm laser as the excitation source.

S7. Theoretical calculations

Density functional theory (DFT) calculations were performed on Gaussian 16 packages at B3LYP/6-31G(d,p) level to determine the HOMO-LUMO energy gap of the ligands in **3**, as well as for DCN molecules.

References

[1] Li, D. *et al.* Highly efficient förster resonance energy transfer between an emissive tetraphenylethylene-based metal–organic cage and the encapsulated dye guest. *Chem. Sci.* **14**, 2237–2244 (2023).

[2] Mu, C. *et al.* Tetraphenylethylene-based multicomponent emissive metallacages as solid-state fluorescent materials. *Angew. Chem. Int. Ed.* **60**, 12293–12297 (2021).

[3] Zhao, S.-S., Chen, L., Wang, L. & Xie, Z. Two tetraphenylethene-containing coordination polymers for reversible mechanochromism. *Chem. Commun.* **53**, 7048–7051 (2017).

[4] Dolomanov, O. V., Bourhis, L. J., Gildea, R. J., Howard, J. A. K. & Puschmann, H. OLEX2: a complete structure solution, refinement and analysis program. *J. Appl. Crystallogr.* **42**, 339–341 (2009).

[5] Sheldrick, G. M. Crystal structure refinement with ıt SHELXL. *Acta Crystallogr. C* **71**, 3–8 (2015).
